# The limitations of using risk factors to screen for blunt cerebrovascular injuries: the harder you look, the more you find

**DOI:** 10.1186/s13017-015-0040-7

**Published:** 2015-09-26

**Authors:** Lewis E. Jacobson, Mary Ziemba-Davis, Argenis J. Herrera

**Affiliations:** Department of Surgery, St. Vincent Indianapolis Hospital, 2001 West 86th Street, Indianapolis, IN 46260 USA; St. Vincent Neuroscience Institute, 8333 Naab Road, Indianapolis, IN 46260 USA

**Keywords:** Blunt cerebrovascular injury, Carotid artery injury, Vertebral artery injury, Blunt trauma, Computer tomography, CT angiography, CTA screening, Risk factors for BCVI, Signs/symptoms of BCVI, Stroke

## Abstract

**Introduction:**

Blunt cerebrovascular injury (BCVI) is reported to occur in 1–2 % of blunt trauma patients. Clinical and radiologic risk factors for BCVI have been described to help identify patients that require screening for these injuries. However, recent studies have suggested that BCVI frequently occurs even in the absence of these risk factors. The purpose of this study was to determine the incidence of BCVI in blunt trauma patients without risk factors and whether these patients could be identified by a more liberal CTA screening protocol.

**Methods:**

We conducted a retrospective cohort study of all blunt trauma patients seen between November 2010 and May 2014. In May 2012, a clinical practice guideline for CTA screening for BCVI was implemented. The records of all patients with BCVI were reviewed for the presence of risk factors for BCVI previously described in the literature.

**Results:**

During the 43 month study period, 6,602 blunt trauma patients were evaluated, 2,374 prior to, and 4,228 after implementation of the clinical practice guideline. Nineteen percent of all blunt trauma patients underwent CTA of the neck after protocol implementation compared to only 1.5 % prior to protocol implementation (*p* = 0.001). As a result, a 5-fold increase in the identification of BCVI was observed (*p* = 0.00003). Thirty-seven percent of patients with BCVI identified with the enhanced CT screening protocol had none of the signs, symptoms, or risk factors usually associated with these injuries.

**Conclusions:**

Our findings demonstrate that reliance on clinical or radiologic risk factors alone as indications for screening for BCVI is inadequate. We recommend routine CTA screening for BCVI in all patients who have sustained a mechanism of injury sufficient to warrant either a CT of the cervical spine or a CTA of the chest.

## Introduction

Although previously thought to be rare, blunt cerebrovascular injury (BCVI) is now reported to occur in 1–2 % of blunt trauma patients [1–3]. Early recognition of these injuries is crucial as the stroke rate in untreated patients with BCVI is reported to be 20–60 % [4–9]. The majority of these strokes occur following a latent, asymptomatic time interval that can vary from hours to weeks [7, 10]. Initiation of treatment with antiplatelet agents or anticoagulation therapy during this asymptomatic period appears to reduce the stroke rate to below 1 % [7, 11, 12].

Screening criteria and optimal imaging modalities to identify patients with BCVI have been vigorously debated over the last 15 years. The groups in Denver [13] and Memphis [1] have identified an extensive list of clinical and radiologic risk factors that warrant diagnostic imaging and these studies have formed the basis of practice management guidelines published by the Western Trauma Association (WTA) in 2009 [13] and the Eastern Association for the Surgery of Trauma (EAST) in 2010 [1]. Despite these extensive lists of risk factors, several groups using whole body multi-slice screening computed tomography (CT) in multiple blunt trauma patients have recognized that as many as 30 % of patients with BCVI have none of these risks factors [14]. In most of these patients, the first sign of an undetected BCVI will be a completed stroke.

Because of our concern that using risk factors alone might fail to identify a significant proportion of patients with BCVI, our group initiated a protocol of routine CT angiography (CTA) of the neck in any blunt trauma patient who was already undergoing CT of the cervical spine (C-spine) and/or CTA of the chest to screen for these injuries. The purpose of this study was to determine the incidence of BCVI in blunt trauma patients in the absence of any of the widely accepted risk factors currently included in published practice management guidelines and whether these patients could be identified by a more liberal CTA screening.

## Methods

St. Vincent Indianapolis Hospital is a 566 bed American College of Surgeons-verified Level II trauma center located in Indianapolis, Indiana. It serves as the receiving trauma center for the 22 hospital St. Vincent Health system within the state and has a fleet of five helicopters to facilitate scene and inter-facility transports. In May of 2012, we initiated a clinical practice guideline for screening of blunt trauma patients for BCVI. All patients evaluated by the trauma service with a mechanism or injury significant enough to warrant a CT of the C-spine and/or a CTA of the chest underwent a CTA of the neck. Patients transferred from outside hospitals who had already undergone CT of the C-spine and/or CTA of the chest were not mandated to undergo routine screening for BCVI due to the increased risk of a second dose of contrast within 24 hours and the additional radiation exposure to the neck. Similarly, emergency department physicians, who evaluated many of the low mechanism/low risk patients initially, were not bound by the routine CTA of the neck screening guideline. In these patients, CTA of the neck was obtained at the discretion of the attending trauma surgeon based on mechanism of injury, signs or symptoms of BCVI, or the presence of known risk factors for BCVI. In these cases, the study was performed immediately. Prior to initiation of the protocol, CTA screening for BCVI was based on the clinical judgment of the trauma surgeon on call guided by the WTA and EAST guidelines available at that time.

### Imaging Protocol

All trauma studies were performed on one of two 64 slice CT scanners, the GE 750 HD or the GE LightSpeed VCT (GE Healthcare, Waukesha, WI). The sequence of the exams and the timing of the contrast injections were optimized to minimize both the radiation and the contrast load delivered to the patient. All trauma patients were positioned supine, head first, with their arms down at their sides. CT scans of the head and face, when indicated, were performed first without IV contrast. Patients then underwent CTA of the neck and CT of the C-spine, acquired during a single run, followed by CTA of the chest (arterial phase) and CT of the abdomen and pelvis (venous phase).

For the CTA of the neck, contrast injection was performed using 60 mL of iohexol 350 at 4 mL per second, followed by 20 mL of 0.9 % sodium chloride. Once contrast was seen entering the aortic arch, scanning was initiated. Images were acquired at 0.625 mm slice thickness and at 0.625 mm intervals (0.625 mm × 0.625 mm). Sagittal images were reformatted at 2 mm × 2 mm and both coronal and sagittal reformats were done in Maximal Intensity Projection (MIP) mode at 10 mm × 2.5 mm.

Images for the CT of the C-spine were obtained during scanning for the CTA of the neck at 0.625 mm × 0.625 mm. Reformats were then done manually with coronal and sagittal images in bone window at 2 mm × 2 mm, sagittal images in standard window at 2 mm × 2 mm and angled axial reformats in bone and standard window at 2.5 mm × 2.5 mm.

For the CTA of the chest, 90 ml of iohexol 350 was used, at an injection rate of 4 mL per second, followed by 20 mL of 0.9 % sodium chloride. Arterial phase images were acquired at 0.625 mm × 0.625 mm. Coronal and sagittal reformats were done at 3 mm × 2 mm as well as coronal and sagittal images in MIP mode at 5 mm × 3 mm.

CT of the abdomen and pelvis (venous phase) was then automatically done 50 seconds after the start of the CTA chest (arterial phase) and images were acquired at 1.25 mm × 0.75 mm with coronal and sagittal reformats at 3 mm × 2 mm.

### Treatment Protocol

All CTA studies of the neck done during the day were read by an attending neuroradiologist. At night an experienced in-house attending CT radiologist provided a preliminary reading and any positive or equivocal studies were reviewed the next morning by the attending neuroradiologist.

Patients with CTA findings suggestive of a BCVI were seen by an attending neurosurgeon or vascular surgeon and complex or equivocal studies were reviewed by an interventional neuroradiologist. Additional imaging with magnetic resonance angiography or repeat CTA was occasionally performed to help differentiate injuries from atherosclerotic disease. Treatment was initiated at the discretion of the attending neurosurgeon or vascular surgeon. Patients with a CTA reading equivocal for BCVI who were not felt to have injuries by the neurosurgeon or vascular surgeon, and for whom no treatment was initiated, were considered to be false positive studies and were excluded.

### Patient Population

This retrospective cohort study was reviewed and approved by our organization’s Institutional Review Board. The trauma registry was used to identify all blunt trauma patients seen at our trauma center between November 1, 2010 and May 31, 2014. Our trauma registry is compliant with the National Trauma Data Standard™ developed by the American College of Surgeons Committee on Trauma for inclusion in the National Trauma Data Bank™. Subjects were divided into two groups based on whether they were seen prior to (pre-protocol) or after (post-protocol) implementation of our routine CTA screening guideline for BCVI which was initiated on May 1, 2012.

Data collection from the registry included age, sex, mechanism of injury, Glasgow Coma Scale (GCS) score [15] in the emergency department (ED), Abbreviated Injury Scale (AIS08) scores [16], Injury Severity Score (ISS) [17], ICD-9 diagnosis codes, whether the patient underwent CTA of the neck, and mortality prior to discharge. All CTA of the neck reports were reviewed individually by the primary author and all studies with findings suggestive of BCVI were identified and the BCVI graded based on the grading scale proposed by Biffl [18]. If a patient had more than one CTA of the neck done, only the initial study was included.

The medical records of all the patients with positive studies were then reviewed for the presence of any of the signs/symptoms or risk factors for BCVI outlined in the new Denver Health Medical Center BCVI screening guideline described by Burlew [11] (Table 1).

**Table 1:** New Denver Health Medical Center BCVI screening criteria [11] and prevalence in post-protocol sample

Screening criteria	Prevalence in post-protocol sample
Signs/symptoms of BCVI	
Potential arterial hemorrhage from neck/nose/mouth	0
Cervical bruit in patients < 50 years old	0
Expanding cervical hematoma	1
Focal neurologic deficit (TIA, hemiparesis, vertebrobasilar symptoms, Horner’s Syndrome)	1
Neurologic deficit inconsistent with head CT scan findings	0
Stroke on CT or MRI	4
Risk factors for BCVI	
High-energy transfer mechanism associated with:	
Displaced mid-face fracture (LeForte II or III)	1
Mandible fracture	3
Complex skull fracture/basilar skull fracture/occipital condyle fracture	5
Closed head injury with diffuse axonal injury and GCS <6	7
Cervical subluxation or ligamentous injury, transverse foramen fracture, any body fracture, any fracture C1–C3	18
Near hanging with anoxic brain injury	1
Clothesline type injury or seat belt abrasion with significant swelling, pain, or altered mental status	0
Traumatic brain injury with thoracic injuries	7
Scalp degloving	3
Thoracic vascular injuries	3
Blunt cardiac rupture	0

### Statistical Analysis

Minitab 17 (State College, PA) was used for statistical analyses. Proportions and means with ranges were used to summarize and compare patient demographics and the prevalence of CTA screening and BCVI in pre- and post-protocol groups. Pearson’s Chi-Square test for independence (χ^2^) and Student’s t test were used to assess differences by study group. Probability (*p*) values associated with Fisher’s exact test are reported for 2 × 2 χ^2^ tables. Yates correction for continuity was used if expected frequencies were less than 5 in 2 × 2 χ^2^ tables. Binary Logistic Regression was used to calculate the odds of BCVI based on GCS and ISS (mild, moderate, and severe).

## Results

During the 43 month study period, 6,602 patients who had sustained blunt trauma were identified, 2,374 prior to implementation of the routine CTA of the neck guideline (pre-protocol) and 4,228 after implementation (post-protocol). Patient demographics are provided in Table 2.

**Table 2:** Demographic characteristics of study populations

	Pre-Protocol		Post-Protocol			No BCVI		BCVI		
	n	%	n	%	*p*	n	%	n	%	*p*
Patients	2374	36.0	4228	64.0		6551	99.2	51	0.8	
Male	1258	53.0	2330	55.1	0.100	3539	54.3	29	57.0	0.7787
Mortality	74	3.1	136	3.2	0.884	202	3.1	8	15.7	0.001
	**Mean**	**Range**	**Mean**	**Range**	***p***	**Mean**	**Range**	**Mean**	**Range**	***p***
Age (years)	54.6	15 to 100	54.6	14 to 100	0.974	54.6	14 to 100	54.0	16 to 89	0.800
GCS in ED	14.1	3 to 15	14.1	3 to 15	0.711	14.1	3 to 15	10.5	3 to 15	0.001
ISS	8.2	1 to 75	8.6	1 to 75	0.052	8.4	1 to 75	23.2	4 to 75	0.001

GCS = Glasgow Coma Scale; ISS = Injury Severity Score

Of the 2,374 pre-protocol patients only 35 (1.5 %) underwent CTA of the neck to evaluate for BCVI whereas post-protocol 802 (19 %) were screened. Pre-protocol there were 5 patients with BCVI identified for an incidence of 0.2 % (Table 3). Post-protocol, 46 patients with BCVI were identified for an overall incidence of 1.1 % in all blunt trauma patients. However, in patients who underwent CTA of the neck, the incidence was 5.7 % (46/802).

**Table 3:** CTA screening and identification of BCVI by study group

	Pre-Protocol		Post-Protocol		
	n	%	n	%	*p*
All patients	2374	36.0	4228	64.0	
CTA of the neck	35	1.5	802	19.0	0.001
BCVI	5	0.2	46	1.1	0.00003

In all, 61 BCVIs were identified in 51 patients. There were 30 common or internal carotid artery injuries and 31 vertebral artery injuries. Forty-two patients had one BCVI, eight patients had 2 injuries and one patient had 3 injuries. The grading of these injuries is outlined in Table 4.

**Table 4:** Grading of BCVIs [18]

Grade	Definition	n	%
I	Luminal irregularity or dissection with <25 % luminal narrowing	34	55.7
II	Dissection or intramural hematoma with ≥25 % luminal narrowing	7	11.5
III	Pseudoaneurysm	6	9.8
IV	Occlusion	14	23.0
V	Transection with free extravasation	0	0.0
	Total	61	100.0

Compared to non-BCVI patients, those with BCVI had significantly greater mechanism of injury as indicated by higher incidences of motor vehicle crashes (54.9 % vs 23.5 %, *p* = 0.001), motorcycle crashes (7.8 % vs 6.7 %, *p* = 0.016), pedestrians hit by car (13.7 % vs 4.3 %, *p* = 0.006), and hangings/strangulations (2 % vs 0.1 %, *p* = 0.045) and lower incidences of falls (21.6 % vs 52.4 %, *p* = 0.001). BCVI patients were also a more severely injured group as indicated by lower initial GCS scores (10.5 vs. 14.1, *p* = 0.001), higher ISS (23.2 vs. 8.4, *p* = 0.001), and higher mortality (15.7 % vs. 3.1 %, *p* = 0.001) (Table 2). As shown in Table 5, the likelihood of BCVI increased with decreasing GCS score in the ED (*p* < 0.005) and increasing ISS (*p* < 0.005). BCVI was 3.5 times more likely in patients with moderate compared to mild GCS scores, and 7.9 times more likely in patients with moderate compared to mild ISS. These odds ratios were 2.6 and 4.8 respectively, as scores increased from moderate to severe. Patients with severe GCS scores and ISS were 9.4 and 38.3 times more likely to have a BCVI than patients with mild scores.

**Table 5:** Prevalence and likelihood of BCVI based on GCS score and ISS

	No BCVI		BCVI		Likelihood of BCVI based on GCS/ISS	Odds ratio [95 % CI]	χ^2^	*p*
	n	%	n	%				
GCS in ED								
Mild	4655	99.4	27	0.6	Moderate vs. Mild	3.5 [1.2:10.2]	38.3	<0.005
Moderate	195	98.0	4	2.0	Severe vs. Moderate	2.6 [0.9:8.0]		
Severe	295	94.9	16	5.1	Severe vs. Mild	9.4 [5.0:17.5]		
ISS								
Mild	4679	99.8	9	0.2	Moderate vs. Mild	7.9 [3.6:17.1]	88.1	<0.005
Moderate	1513	98.5	23	1.5	Severe vs. Moderate	4.8 [2.6:9.0]		
Severe	258	93.1	19	6.9	Severe vs. Mild	38.3 [17.2:85.5]		

GCS = Glasgow Coma Scale Score: Mild 14–15, Moderate 8–13, Severe < 8

ISS = Injury Severity Score: Mild 1–9, Moderate 10–25, Severe > 25

Treatment of patients with BCVI was tailored to the severity of the BCVI and the patient’s other injuries. Thirty-five patients were treated with antiplatelet agents, 5 with systemic anticoagulation, and 4 with a combination of both. Seven patients received no treatment either because they were too critical or because they died before treatment could be initiated.

Of the 46 post-protocol patients with BCVI, 29 (63 %) had at least one of the signs/symptoms or risk factors outlined in the new Denver Health Medical Center BCVI screening guideline [11] (Table 1). Seventeen patients had a single sign/symptom or risk factor, seven patients had 2, two patients had 3, two patients had 4, and one patient had 6 of the new Denver screening criteria. Cervical spine injury (n = 18) was the most common risk factor, followed by closed head injury with diffuse axonal injury and GCS <6 (n = 7) and traumatic brain injury with thoracic injury (n = 7). Finally, 17 (37 %) of the 46 post-protocol patients with BCVI had no identifiable signs/symptoms or risk factors outlined in the new Denver screening guidelines.

## Discussion

**Fig. 1:**
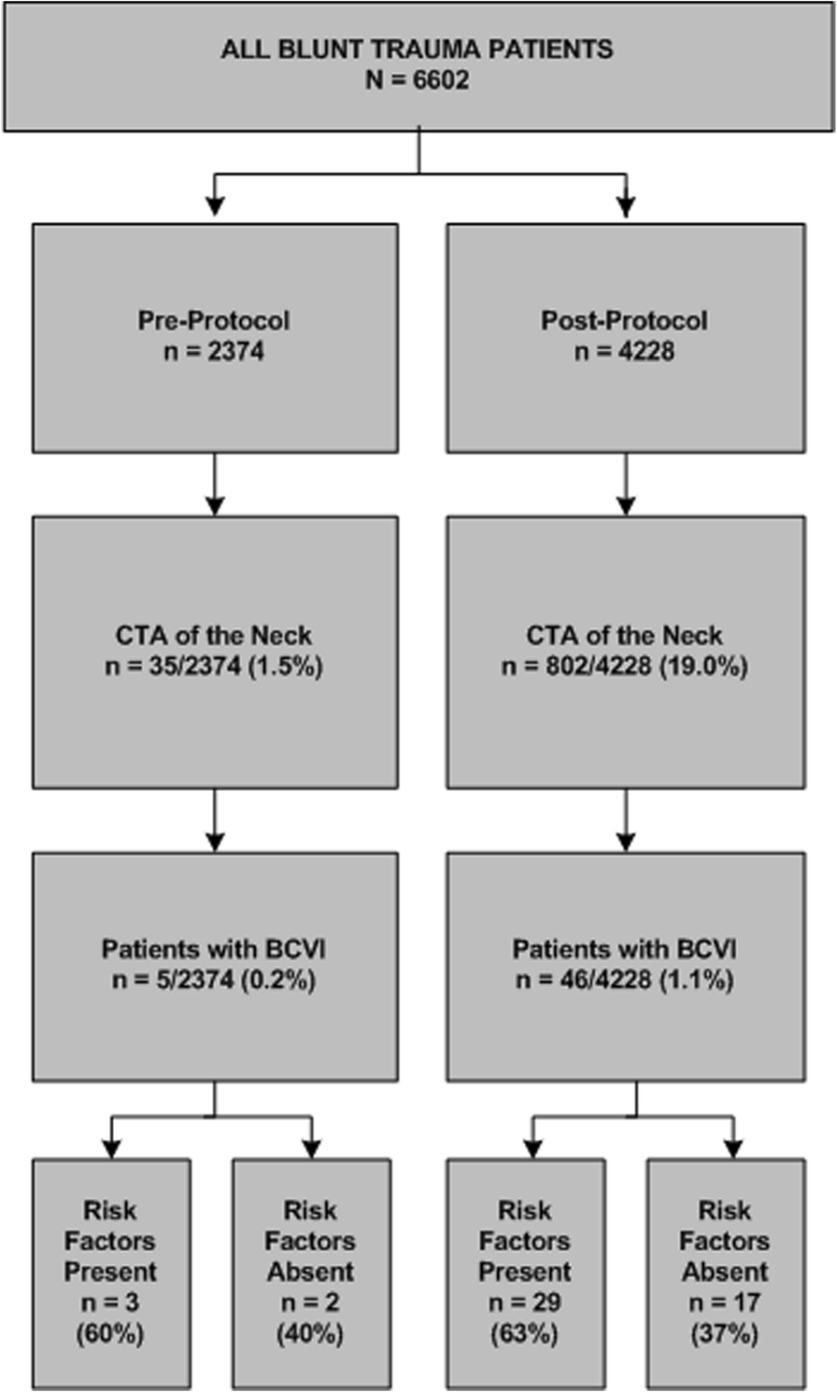
The limitations of clinical and radiologic risk factors to screen for BCVI

In 2012, we initiated a protocol of routine CTA of the neck in any blunt trauma patient who was already undergoing CT of the C-spine and/or CTA of the chest. The purpose of this study was to determine whether identification of patients with BCVI was improved by this CT screening protocol. Pre-protocol only 1.5 % of blunt trauma patients underwent CTA of the neck to screen for BCVI, while post-protocol 19 % were screened (*p* = 0.001), representing a 13-fold increase in CTA screening (Fig. 1). As a result, a 5-fold increase in the incidence of BCVI was identified in our patient population (5/2,374 patients, 0.2 % vs 46/4,228 patients, 1.1 %; *p* = 0.001). This apparent increase in the incidence of BCVI is almost certainly the result of more intensive screening rather than a true increase in incidence. In all likelihood, these injuries were being missed prior to the implementation of our BCVI screening guideline.

Thirty-seven percent of BCVI patients identified with this enhanced CT screening protocol had none of the clinical or radiologic risk factors listed in the expanded Denver screening guidelines [11]. It therefore seems clear that reliance on risk factors alone as the indication for BCVI screening is likely to result in missed injuries and potentially avoidable strokes.

Blunt cerebrovascular injuries are thought to result from a combination of stretch induced disruption of the layers of the vessel wall and direct injury caused by fractures of the transverse foramina of the cervical vertebrae [2]. Identification of these lesions and treatment with anticoagulation or antiplatelet agents is recognized to be crucial to prevent embolization or propagation of thrombus into the distal cerebral vessels.

Until relatively recently, blunt cerebrovascular injury was thought to be a rare entity. In 1990, Davis et al. [19] reported on a series of 15,935 blunt trauma patients admitted over a 5 year period in San Diego County and identified 14 patients with blunt carotid injuries for a detected incidence of 0.08 %. Consistent with the standard of care at that time, all injuries were diagnosed by angiography and the majority (11/14, 79 %) only after symptoms or CT findings of completed stroke. Over the next 20 years, however, several crucial observations significantly altered the management and prognosis of these lesions.

Firstly, anticoagulation was shown to improve neurologic outcomes in patients with strokes related to BCVI [2, 5, 6, 9, 11, 19, 20]. In addition, early anticoagulation or antiplatelet therapy has been shown to decrease stroke rate in asymptomatic patients with BCVI from as high as 60 % to less than 1 % [7, 11, 12, 21]. Full anticoagulation is frequently contraindicated in blunt trauma patients due to associated injuries. A recent Cochrane review [22], however, failed to demonstrate a difference in efficacy between anticoagulation and aspirin and concluded that aspirin was as effective as anticoagulation, but with a lower risk of hemorrhage. This would support early initiation of effective therapy in the form of aspirin, even in high risk trauma patients.

Secondly, it has been widely recognized that the vast majority of BCVIs are asymptomatic at the time of presentation and that neurologic symptoms only develop after some variable time interval. This latent, asymptomatic period prior to development of stroke can vary from minutes to years but most commonly lasts from 10 to 72 hours following injury [2, 4, 6–8, 11, 18, 23–26]. Although patients may occasionally present with symptoms of transient ischemic attack, in most the first symptom will be a completed ischemic stroke. It is therefore crucial to identify BCVI during this latent period and to initiate treatment before irreversible neurologic injury occurs.

This recognition led to the third important discovery that has reduced the morbidity and mortality of these lesions, namely the identification of risk factors in blunt trauma patients that warrant screening for BCVI prior to the development of neurologic sequelae. By the late 1990’s it was clear that there was an unrecognized epidemic of BCVI [20]. In a meta-analysis done by Franz et al. [2] the incidence range was 0.45–1.63 % in studies using 16-slice or greater CTA. At the high end, this is 20 times higher than the incidence in Davis’ study in 1990 [19] of 0.08 %. Recognizing that most of these injuries are asymptomatic at the time of presentation, the groups in Denver [11, 13, 21] and Memphis [1, 27–29] have, over the last decade, elucidated an extensive list of clinical and radiologic risk factors that warrant screening for BCVI. Publication of practice management guidelines by the WTA in 2009 [13] and EAST in 2010 [1] led to widespread adoption of these screening criteria for BCVI. However, despite subsequent broadening of the list of risk factors, it has been recognized that at least 20–30 % of patients with BCVI have none of the risk factors for screening outlined in these organizational guidelines or other published research [9, 14, 28].

In a study by Emmett et al. [28], most patients with significant blunt trauma who warranted a head, C-spine, or face CT to evaluate for potential injury underwent CTA of the neck at the time of their initial trauma evaluation. They found that this routine screening with CTA identified that 16 % of their patients with BCVI had none of the conventional risk factors for screening. Further confirmation of the lack of sensitivity of these widely used risk factors as screening criteria for BCVI has been published by the group from Baltimore [9, 14]. Since 2004 they have used a whole-body, multi-detector CT screening protocol for blunt trauma patients clinically judged to be at high risk for significant injury. In a study published in 2014 they found that 30 % of patients diagnosed with BCVI using this technique had none of the radiologic or clinical risk factors previously described for BCVI screening [14]. They concluded that the use of currently available risk factors to identify patients for screening would lead to missed injury and stroke and that more liberalized screening for BCVI during initial whole-body CT imaging based on mechanism alone is warranted.

Based on our analysis of the literature, we had come to a similar conclusion, even prior to publication of the Baltimore study [14], and initiated a guideline for routine CTA of the neck in patients undergoing CT of the C-spine and/or CTA of the chest. This conclusion is now supported by the finding in this study that 37 % of patients with BCVI had none of these widely accepted risk factors.

Although 4-vessel digital subtraction angiography (DSA) had long been considered the gold standard for the diagnosis of BCVI, most trauma surgeons do not currently consider it to be the preferred method for screening patients for these injuries. The technique is invasive, expensive, labor intensive, and continues to have a small but measurable potential for complications, including stroke [2, 29]. In addition, it may not be available outside of high volume trauma centers and tertiary care hospitals [9, 14]. CTA, in comparison, is widely available, non-invasive, rapid and cost effective and can be used to detect other injuries in the neck with a single imaging series. Although earlier studies cautioned against use of CTA to identify BCVI due to inadequate sensitivity, more recent data from studies using multi-slice CT scanners have demonstrated improved sensitivity allowing recommendation of its use for screening [29–31]. Moreover, based on a recent survey, the use of CTA of the neck seems to be widespread, with 93 % of 137 trauma surgeons reporting CTA as their preferred method of imaging for the diagnosis of BCVI [32].

There are several limitations to this study. The intent of our screening protocol was to order a CTA of the neck in any patient with sufficient mechanism or clinical suspicion of injury to warrant imaging with CT of the C-spine and/or CTA of the chest. However, only 19 % of post-protocol blunt trauma patients underwent CTA of the neck. This resulted from several factors. A significant number of low mechanism and low risk patients were not felt to warrant either CT of the C-spine or CTA of the chest and therefore did not undergo CTA of the neck. In addition, CTA of the neck was not mandated in the absence of risk factors in those patients who had already undergone CT of the C-spine and/or CTA of the chest, either prior to transfer from an outside hospital or prior to consultation by the trauma service. Nevertheless, institution of this guideline resulted in a 13-fold increase in the number of CTAs of the neck obtained and a 5-fold increase in the percentage of patients in whom BCVI was identified. Given the high risk of undiagnosed and untreated BCVI reported in the literature, identification of these additional injuries by a liberal screening guideline, and subsequent treatment, almost certainly reduced the number of strokes in these patients. Furthermore, although the incidence of BCVI in all post-protocol patients was 1.1 %, the incidence in the 19 % of patients who were screened with CTA of the neck was 5.7 %. It is likely that more intensive screening would yield an even higher incidence of BCVI. For instance, approximately 40 % of our patients were transferred from outside hospitals and most of them had undergone CT imaging prior to transfer and were therefore not mandated to undergo CTA of the neck in the absence of risk factors. Based on our results we plan to recommend our clinical practice guideline for BCVI screening to the 22 hospitals in our system as well as other referring hospitals, and to implement it within our emergency department physician group for patients seen prior to consultation by the trauma service.

The optimal strategy for identification of BCVI continues to evolve. CTA now appears to be widely accepted as the screening study of choice and given its low risk, ubiquitous availability and reasonable cost in comparison to angiography, it could even be considered the new gold standard. Currently available screening guidelines fail to identify more than a third of patients with BCVI and should therefore no longer be considered adequate by themselves for this purpose. In patients undergoing CT of the C-spine and/or CTA of the chest, a dedicated CTA of the neck can be obtained with no increase in radiation exposure and minimal increase in the amount of contrast administered. In addition, this technique of routine, simultaneous CTA of the neck in moderate and high risk blunt trauma patients obviates the need for a return trip to the CT scanner and additional radiation and contrast in patients who have risk factors identified on initial imaging. This allows the earliest possible identification of these potentially devastating injuries and initiation of simple, low risk treatment (such as aspirin) which appears to reduce the stroke rate to less than 1 % [5, 11, 21, 22]. Based on the findings of this study we would recommend routine CTA of the neck in all patients who have sustained a mechanism of injury sufficient to warrant either a CT of the C-spine or a CTA of the chest. This should minimize the risk of missing occult BCVI in patients with these injuries who have none of the clinical or radiologic risk factors identified in currently available clinical screening guidelines.
